# Higher soluble TREM-1 levels are associated with cognitive impairment after acute ischemic stroke

**DOI:** 10.3389/fnagi.2024.1463065

**Published:** 2024-11-22

**Authors:** Zhuo Chen, Xin Yi, Wei Fu, Yong Wu, Xingju Zhong, Chaoli Fan, Yu Jiang, Qi Zhou, Jie Peng, Jieyu Liao, Zhike You, Jingyu Tan

**Affiliations:** ^1^Department of Neurology, Mianzhu People’s Hospital, Mianzhu, Sichuan, China; ^2^Department of Endocrinology, Mianzhu People’s Hospital, Mianzhu, Sichuan, China

**Keywords:** biomarker, prediction, triggering receptor expressed on myeloid cells-1, stroke, cognitive impairment

## Abstract

**Background and purpose:**

Triggering receptor expressed on myeloid cells-1 (TREM-1) was reported to be critical for mediating the neurological function after stroke, while the impact of soluble TREM-1 (sTREM-1) on cognitive impairment after ischemic stroke is unclear. We aimed to explore the association between sTREM-1 and post-stroke cognitive impairment (PSCI).

**Methods:**

We prospectively recruited consecutive ischemic stroke patients who admitted hospital within 7 days of onset. Serum sTREM-1 concentrations were measured after admission. Cognitive function was assessed at 90 days follow-up using the Mini-Mental State Examination (MMSE) and Montreal Cognitive Assessment (MoCA). PSCI was defined as a MMSE score of <27 or a MoCA score < 26.

**Results:**

A total of 291 patients (mean age, 66.6 years; 46.0% female) were enrolled for this study. Among these participants, the median sTREM-1 concentrations were 289.4 pg/mL. According to the MoCA score, 153 (52.6%) patients experienced PSCI at 3 months. After adjustment for confounding risk factors by multivariate regression analysis, patients with sTREM-1 levels in the fourth quartile were more likely to have increased risk 3-month PSCI (as compared with the first quartile, odds ratio 12.22, 95% confidence intervals, 5.20–28.71, *P* = 0.001). Restricted cubic spline further confirmed a dose-dependent relationship between sTREM-1 levels and PSCI (*P* = 0.003 for linearity). Similar significant findings were observed when the cognitive impairment was diagnosed according to the MMSE criterion.

**Conclusion:**

Our study revealed that higher serum sTREM-1 levels at admission were associated with increased risk of 3-month PSCI.

## Introduction

Ischemic stroke is a prevalent cerebrovascular disease and a major cause of mortality and long-term morbidity throughout the world (Feigin et al., 2014; Giroud et al., 2014). Post-stroke cognitive impairment (PSCI) is recognized as one of the most common complications after stroke, occurring in one half of stroke survivors (Barbay et al., 2018a; Barbay et al., 2018b). There is evidence that PSCI is an independent predictor of functional disability, as well as higher mortality and recurrent stroke risk (Melkas et al., 2009; Kjörk et al., 2016; Yaghi et al., 2020). Early identification of biomarkers for predicting PSCI may have clinical implications for better prevention, and treatment of the disease.

The triggering receptor expressed on myeloid cells-1 (TREM-1) is an immune receptor initially known to be expressed on neutrophils and monocytes (Bouchon et al., 2000). It is involved in the amplification of the innate immune response through synergizing with toll-like receptor in infectious and non-infectious diseases (Bouchon et al., 2001; Colonna and Facchetti, 2003). In recent studies, it has been shown that circulating soluble TREM-1 (sTREM-1) plays a critical role in cerebrovascular diseases, such as subarachnoid hemorrhage, in-stent restenosis, and cardiovascular events (Sun et al., 2017; Wang et al., 2017; Wang et al., 2018). Experimental data showed that LP17 targeting TREM-1 may attenuate cerebral ischemia-induced neuronal damage by inhibiting oxidative stress and pyroptosis (Liang et al., 2020). Furthermore, blockade of TREM-1 can improve long-term functional outcomes in the hippocampus by alleviating cellular proliferation and synaptic plasticity (Xu et al., 2019). Considering that TREM-1 exerts a detrimental effect on neurological function after ischemic stroke, there might be a potential correlation between circulating sTREM-1 levels and PSCI. Therefore, our study prospectively investigated whether serum sTREM-1 concentrations in acute phase were associated with cognitive impairment at 3 months after ischemic stroke in a cohort of Chinese patients.

## Materials and methods

### Study design and population

In the present study, first-time ischemic stroke patients within 7 days of the onset of symptoms were consecutively screened for eligibility at Mianzhu People’s Hospital between January 2023 and August 2023. The exclusion criteria were as follows: (1) age ≤ 18 years old; (2) patients with pre-existing cognitive impairment, such as Alzheimer’s disease, Parkinson’s disease, and other neurodegenerative diseases; (3) patients with severe neurological deficits, which impeded the neuropsychological testing; (4) patients with any history of central nervous system disease, severe hepatic or renal disease, autoimmune disease, or thyroid disorders. We also excluded the patients with a life expectancy < 3 months. The study was approved by the ethics committee of the Mianzhu Hospital and written informed consent was obtained from each patient.

### Data collection

Data collection was conducted using a standardized case report form after admission. For each patient we recorded: demographic data (age, gender, and education); vascular risk factors (hypertension, diabetes, smoking, dyslipidemia, and coronary artery disease); clinical data (medication history, blood pressure, stroke severity, and stroke etiology); laboratory data (lipid profile, fasting blood-glucose, high-sensitivity C-reactive protein and sTREM-1 levels). Baseline stroke severity was assessed by certified neurologist using National Institutes of Health Stroke Scale (NIHSS) (Goldstein and Samsa, 1997). Stroke subtype was classified basing on the criteria of Trial of Org 10172 in Acute Stroke Treatment (Adams et al., 1993). The infarction volume was assessed by the semiquantitative DWI-Alberta Stroke Program Early CT Score (DWI-ASPECTS), which is increasingly used in clinical settings (Lassalle et al., 2016).

### sTREM-1 concentrations measurement

The blood samples were analyzed a laboratory technician who blinded to the clinical data. Blood samples were obtained from each subject within 24 h after admission. The specimens were centrifuged at 2500 g for 15 min and the isolated serum frozen at −80°C for further analysis. sTREM-1 concentrations were measured by enzyme-linked immunosorbent assay (R&D Systems, Minneapolis, MN). The operation was carried out according to the specification.

### Cognitive function measurement

A cognitive function evaluation was performed by neurologists blinded to clinical and laboratory data at 3-months after stroke onset, using the Mini-Mental State Examination (MMSE) and Montreal Cognitive Assessment (MoCA). 77 tool for assessing cognitive impairment in Chinese population. In this study, PSCI was defined as a MMSE score of <27 (Webb et al., 2014; Zhong et al., 2018) or a MoCA score < 26 (Campbell et al., 2016; Geng et al., 2017). Considering the influence of education, 1 point was added for patients with education < 12 years on the total MoCA score (Nasreddine et al., 2005).

### Statistical analysis

Data normality was determined using the Kolmogorov-Smirnov test. Normally distributed continuous variables are presented as means and were compared using Student’s *t*-test and one-way analysis of variance. Not normally distributed variables were presented as median (interquartile range) and were compared using Mann–Whitney U test and Kruskal-Wallis test. Categorical variables are expressed as percentage and were compared using χ^2^ test and Fisher exact test. Multiple logistic regression analysis was used to evaluate whether increased sTREM-1 levels were associated with the presence of PSCI. Model 1 was adjusted for age and sex. Model 2 was adjusted for age, sex and the variables with a *P*-value < 0.1 in the univariate analysis. Odds ratios (OR) and 95% confidence intervals (CI) were calculated.

Restricted cubic spline was utilized to detect the possible linear dependency of relationship between the risk of PSCI and sTREM-1 levels, using 4 knots chosen at the 5th, 35th, 65th, and 95th percentiles.

Furthermore, receiver operating characteristic (ROC) curves were applied to investigating the accuracy of different models in predicting PSCI. The Z test was used to compare the area under the curve (AUC) of different models. A *P-*value < 0.05 at two-tailed was considered statistically significant. All statistical analyses were performed on SPSS for Windows, version 24.0 (SPSS Inc., Chicago, IL, USA) and R 3.6.0.

## Results

We included a total of 291 stroke patients (mean age, 66.6 ± 9.2 years), which consisted of 157 males (54.0%) and 134 females (46.0%). Their median levels of sTREM-1 were 289.4 pg/mL. We divided all patients into 4 groups according to the quartiles of sTREM-1 levels: first quartile (<224.2 pg/mL); second quartile (224.2–287.4 pg/mL); third quartile (287.5–388.7 pg/mL); and fourth quartile (>388.7 pg/mL). Table 1 demonstrated the demographic characteristics, clinical data and laboratory data according to the quartiles of sTREM-1 levels. Age, hypertension, total cholesterol levels and high-sensitivity C-reactive protein levels differed significantly with increasing quartiles of sTREM-1.

**TABLE 1:** Baseline characteristics of the study subjects stratified by sTREM-1 quartile.

Variables	sTREM-1 quartile				*P-*value
	**First, *n* = 71**	**Second, *n* = 74**	**Third, *n* = 73**	**Fourth, *n* = 73**	
Age, year	65.6 ± 9.4	64.5 ± 9.4	68.1 ± 8.7	68.2 ± 8.8	0.029
Female, *n* (%)	30 (42.3)	30 (40.5)	35 (47.6)	39 (53.4)	0.389
Education < 12 years, n (%)	46 (63.4)	48 (64.9)	51 (69.9)	40 (54.8)	0.296
**Vascular risk factors, n (%)**					
Hypertension	33 (46.5)	42 (56.8)	41 (56.2)	51 (69.9)	0.043
Diabetes mellitus	18 (25.4)	19 (25.7)	23 (31.5)	16 (21.9)	0.616
Hyperlipidemia	13 (18.3)	10 (13.5)	11 (15.1)	9 (12.3)	0.765
Coronary heart disease	7 (9.9)	9 (12.2)	8 (11.0)	10 (13.7)	0.903
Current smoking	26 (36.6)	27 (36.5)	29 (39.7)	25 (34.2)	0.924
**Clinical data**					
Previous antiplatelet, *n* (%)	18 (25.4)	22 (29.7)	23 (31.5)	23 (31.5)	0.823
Previous statin, *n* (%)	21 (29.6)	19 (25.7)	17 (23.3)	18 (24.7)	0.845
Previous antihypertensive, *n* (%)	22 (31.0)	25 (33.8)	23 (31.5)	19 (26.0)	0.776
Onset-to-blood drawing time, day	3.0 (1.0, 4.0)	3.0 (2.0, 4.0)	2.0 (1.0, 4.0)	3.0 (2.0, 4.0)	0.408
NIHSS, score	4.0 (3.0, 7.0)	5.0 (2.0, 6.5)	5.0 (2.0, 8.0)	5.0 (3.0, 8.0)	0.205
White matter lesions, *n* (%)	31 (43.7)	32 (43.2)	25 (34.2)	27 (37.0)	0.576
DWI-ASPECTS 0–7, *n* (%)	23 (35.4)	31 (44.9)	32 (45.7)	30 (44.8)	0.586
Systolic blood pressure, mmHg	139.4 ± 15.3	139.8 ± 18.8	135.9 ± 16.4	136.5 ± 15.7	0.366
Diastolic blood pressure, mmHg	81.6 ± 10.6	82.5 ± 10.8	80.0 ± 9.7	79.4 ± 8.8	0.166
Stroke subtypes, *n* (%)					0.646
Large artery atherosclerosis	31 (43.7)	33 (44.6)	35 (47.9)	29 (39.7)	
Cardioembolism	13 (18.3)	14 (18.9)	12 (16.4)	19 (26.0)	
Small artery occlusion	17 (23.9)	23 (31.1)	21 (28.8)	19 (26.0)	
Others	10 (14.1)	4 (5.4)	5 (6.8)	6 (8.2)	
**Laboratory data**					
Total cholesterol, mmol/L	3.7 ± 0.9	4.0 ± 1.1	4.4 ± 1.1	4.5 ± 1.2	0.004
Triglyceride, mmol/L	1.5 (0.8, 1.8)	1.4 (1.1, 1.8)	1.3 (0.9, 1.6)	1.3 (1.0, 1.9)	0.392
Low-density lipoprotein, mmol/L	2.3 (1.8, 2.7)	2.3 (1.8, 2.8)	2.6 (3.1, 3.2)	2.4 (2.0, 2.9)	0.151
High-density lipoprotein, mmol/L	1.0 ± 0.2	1.1 ± 0.2	1.1 ± 0.2	1.1 ± 0.3	0.213
Hs-CRP, mg/L	4.7 (2.3, 9.7)	4.9 (2.3, 9.3)	5.8 (2.8, 9.6)	8.4 (4.1, 12.2)	0.026
Neutrophil-to-lymphocyte ratio	6.9 (4.3, 9.3)	7.6 (4.5, 10.7)	7.3 (4.1, 13.2)	7.9 (5.2, 11.0)	0.289
Fasting blood glucose, mmol/L	5.8 ± 2.5	5.6 ± 2.0	6.0 ± 2.5	6.3 ± 3.0	0.376

DWI-ASPECTS, DWI based Alberta stroke program early CT score; Hs-CRP, hyper-sensitive C-reactive protein; NIHSS, National Institutes of Health Stroke Scale; sTREM-1, soluble triggering receptor expressed on myeloid cells-1.

**FIGURE 1:**
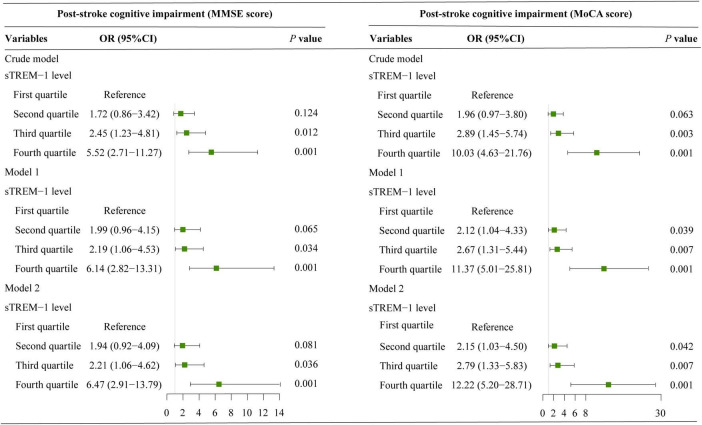
Binary logistic regression models explore the association between serum sTREM-1 levels and risk of PSCI. Model 1 adjusted for age and sex; model 2 adjusted age, sex and variables with a *P*-value < 0.1 in univariate analysis.

**FIGURE 2:**
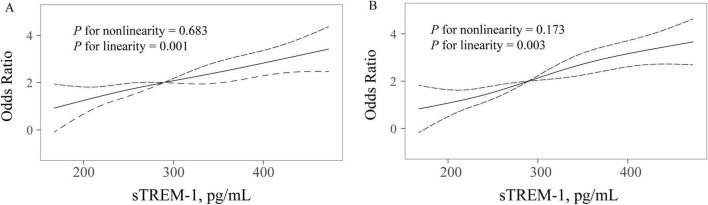
Restricted cubic spline detects the possible linear dependency of relationship between sTREM-1 levels and risk of PSCI (**A** for MMSE category; **B** for MoCA category). Restricted cubic spline of odds ratios and 95% confidence intervals with knots located at the 5th, 35th, 65th and 95th percentiles of the distribution of sTREM-1. The midpoint of sTREM-1 serves as the reference point. Odds ratios were adjusted for the same covariates in model 2.

Results of univariate analysis between patients with and without PSCI were showed in Table 2. According to MoCA category, 153 patients (52.6%) were diagnosed as PSCI. Univariate analysis showed that participants with PSCI were older, had higher baseline NIHSS score and fasting blood glucose levels, and were more likely to have hypertension, diabetes mellitus, white matter lesions and education < 12 years. According to the MMSE category, 140 patients (48.1%) experienced PSCI at 3 months. Patients with PSCI were older, had higher high-sensitivity C-reactive protein levels, and were more likely to have hypertension, diabetes mellitus and education < 12 years. After adjustment for age, sex, education years, and variables with *P*-value < 0.1 in univariate analysis, multivariate regression model demonstrated that patients with sTREM-1 levels in the fourth quartile were more likely to have increased risk 3-month PSCI (OR 12.22, 95% CI, 5.20–28.71, *P* = 0.001 for MoCA category; OR 6.47, 95% CI, 2.91–13.79, *P* = 0.001 for MMSE category), as compared with the first quartile (Figure 1). Restricted cubic spline further confirmed a dose-dependent relationship between sTREM-1 levels and PSCI (*P* = 0.003 for linearity for MoCA category; *P* = 0.001 for linearity for MMSE category; Figure 2). We also confirmed a negative association of sTREM-1 levels with MMSE score (as continuous variable, Spearman’s Rho coefficient = −0.346, *P* = 0.001) and MoCA score (as continuous variable, Spearman’s Rho coefficient = −0.335, *P* = 0.001).

**TABLE 2:** Baseline characteristics according to the participants with and without PSCI.

Variables	PSCI (MMSE)			PSCI (MoCA)		
	**Presence, *n* = 140**	**Absence, *n* = 151**	***P*-value**	**Presence, *n* = 153**	**Absence, *n* = 138**	***P*-value**
Age, year	69.3 ± 8.3	64.1 ± 9.1	0.001	68.5 ± 8.6	64.5 ± 9.4	0.001
Female sex, *n* (%)	69 (49.3)	65 (43.0)	0.286	73 (47.7)	61 (44.2)	0.549
Education < 12 years, *n* (%)	84 (55.6)	100 (71.4)	0.005	106 (69.3)	78 (56.5)	0.024
**Cardiovascular risk factors, n (%)**						
Hypertension	90 (64.3)	77 (51.0)	0.022	98 (64.1)	69 (50.0)	0.015
Diabetes mellitus	44 (31.4)	32 (21.2)	0.047	48 (31.4)	28 (20.3)	0.032
Hyperlipidemia	20 (14.3)	23 (15.2)	0.820	21 (13.7)	22 (15.9)	0.595
Coronary heart disease	17 (12.1)	17 (11.3)	0.814	17 (11.1)	17 (12.3)	0.749
Current smoking	52 (37.1)	55 (36.4)	0.899	60 (39.2)	47 (34.1)	0.362
**Clinical data**						
Previous antiplatelet, *n* (%)	44 (31.4)	42 (27.8)	0.501	48 (31.4)	38 (27.5)	0.474
Previous statin, *n* (%)	38 (27.2)	37 (24.5)	0.607	42 (27.5)	33 (23.9)	0.491
Previous antihypertensive, *n* (%)	40 (28.6)	49 (32.5)	0.473	41 (26.8)	48 (34.8)	0.140
NIHSS, score	5.0 (3.0, 8.0)	5.0 (2.0, 7.0)	0.568	5.0 (3.0, 8.0)	4.5 (2.0, 6.0)	0.007
White matter lesions, *n* (%)	63 (45.0)	52 (34.4)	0.066	69 (45.1)	46 (33.3)	0.042
DWI-ASPECTS 0–7, *n* (%)	61 (47.3)	55 (38.7)	0.155	66 (46.5)	50 (38.8)	0.200
Systolic blood pressure, mmHg	136.9 ± 15.4	138.9 ± 17.6	0.311	136.4 ± 15.3	139.5 ± 17.8	0.112
Diastolic blood pressure, mmHg	80.3 ± 8.9	81.1 ± 11.0	0.508	79.8 ± 9.1	81.7 ± 10.9	0.115
Stroke subtypes, *n* (%)			0.671			0.255
Large artery atherosclerosis	59 (42.1)	69 (45.7)		64 (41.8)	64 (46.4)	
Cardioembolism	32 (22.9)	26 (17.2)		37 (24.2)	21 (15.2)	
Small artery occlusion	38 (27.1)	42 (27.8)		41 (26.8)	39 (28.3)	
Others	11 (7.9)	14 (9.3)		11 (7.2)	14 (10.1)	
**Laboratory data**						
Total cholesterol, mmol/L	4.3 ± 1.2	4.1 ± 1.1	0.212	4.3 ± 1.2	4.1 ± 1.1	0.109
Triglyceride, mmol/L	1.5 (1.0, 1.8)	1.3 (1.0, 1.8)	0.548	1.4 (1.0, 1.8)	1.4 (1.0, 1.8)	0.711
Low-density lipoprotein, mmol/L	2.4 (1.9, 2.7)	2.4 (2.0, 3.1)	0.201	2.4 (2.0, 2.9)	2.3 (2.0, 3.1)	0.443
High-density lipoprotein, mmol/L	1.1 ± 0.2	1.1 ± 0.3	0.992	1.1 ± 0.2	1.1 ± 0.2	0.509
Hs-CRP, mg/L	7.4 (3.3, 10.5)	5.5 (2.4, 9.7)	0.026	6.8 (3.3, 10.3)	5.6 (2.4, 9.7)	0.134
Neutrophil-to-lymphocyte ratio	7.4 (5.1, 11.2)	7.2 (4.2, 10.5)	0.143	7.3 (5.1, 11.0)	7.2 (4.1, 10.5)	0.212
Fasting blood glucose, mmol/L	6.0 ± 2.5	5.8 ± 2.4	0.480	6.0 ± 2.5	5.8 ± 2.5	0.386
sTREM-1 level, (pg/mL)	339.1 (256.9, 409.3)	245.4 (212.3, 325.3)	0.001	346.8 (260.9, 409.9)	236.8 (209.8, 309.5)	0.001
sTREM-1 quartile, *n* (%)			0.001			0.001
First quartile	21 (15.0)	50 (33.1)		21 (13.7)	50 (36.2)	
Second quartile	31 (22.1)	43 (28.5)		33 (21.6)	41 (29.7)	
Third quartile	37 (26.4)	36 (23.8)		40 (26.1)	33 (23.9)	
Fourth quartile	51 (36.4)	22 (14.6)		59 (38.6)	14 (10.1)	

Hs-CRP, hyper-sensitive C-reactive protein; NIHSS, National Institutes of Health Stroke Scale; PSCI, post-stroke cognitive impairment; sTREM-1, soluble triggering receptor expressed on myeloid cells-1.

**FIGURE 3:**
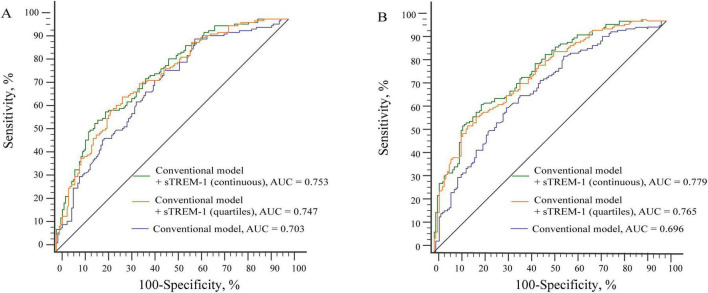
Receiver operating characteristic compares the potential of different models in predicting PSCI (**A** for MMSE category; **B** for MoCA category). **(A)** The AUC was increased from 0.703 to 0.753 when sTREM-1 was put into the conventional model. Conventional model included age, education, hypertension, diabetes mellitus and high-sensitivity C-reactive protein levels. **(B)** The AUC was increased from 0.696 to 0.779 when sTREM-1 was put into the conventional model. Conventional model included age, education, hypertension, diabetes mellitus, white matter lesions, baseline NIHSS score and fasting blood glucose levels.

We further investigated whether adding serum sTREM-1 levels to the conventional risk factors could improve the risk prediction of PSCI. As shown in Figure 3, the AUC for predicting PSCI was increased (from 0.696 to 0.779, *P* = 0.001 for MoCA category; from 0.703 to 0.753, *P* = 0.002 for MMSE category) when sTREM-1 was put into the model. Similar results were found when sTREM-1 was analyzed as a categorical variable.

## Discussion

In this study using a prospective cohort, we examined the association between serum sTREM-1 levels on admission and 3-month PSCI. Our results indicated that higher serum sTREM-1 levels were independently associated with cognitive impairment following acute ischemic stroke, regardless of age, gender, degree of education or other known risk factors. There is a wide range of cognitive impairment after stroke, ranging from 20 to 80% (Sun et al., 2014). The variation in prevalence is mainly due to the difference in definition of PSCI, the interval since stroke onset, study populations, and study methods. Using the MoCA category, 52.6% of stroke patients presented with PSCI in this study, which is similar to previous meta-analysis (Barbay et al., 2018a).

According to our results, PSCI patients at 3 months had a significantly higher NIHSS score than patients without PSCI, in line with previous studies (Melkas et al., 2009; Geng et al., 2017). The patients from PSCI group were also more likely to have diabetes mellitus. There are several mechanisms by which hyperglycemia can impair cognitive function, including advanced glycation end-products, inflammation, and microvascular disease (Yaffe et al., 2012). Furthermore, PSCI was also more prevalent in patients with white matter lesions, which was also consistent with previous study (Zhang et al., 2017; Khan et al., 2019). The reason for this is likely to be caused by loss of microstructural integrity in white matter tracts, which prevents structural reorganization after a stroke and reduces functional compensation through remote areas of the brain (Della Nave et al., 2007; Pantoni, 2010; Grefkes and Fink, 2014). It has been reported that proinflammatory factors play an important role in PSCI in previous studies (Narasimhalu et al., 2015). However, there were no significant differences in levels of high-sensitivity C-reactive protein between PSCI and non-PSCI groups, which was potentially due to the different definitions of PSCI.

The TREM-1 immune receptor amplifies the innate immune response by expressing itself on myeloid cells (Bouchon et al., 2001; Colonna and Facchetti, 2003). The circulating form of TREM-1 arises from spliced TREM-1 on neutrophils, macrophages, and mature monocyte membranes. Experimental data have demonstrated that the upregulation of neutrophil and monocyte membrane TREM-1 during endotoxemia is associated with an elevated release of sTREM-1 in the blood (Gibot et al., 2004). This process also occurs in various cerebrovascular diseases including subarachnoid hemorrhage, in-stent restenosis and cardiovascular events (Sun et al., 2017; Wang et al., 2017; Wang et al., 2018). Patients with early post-stroke depressive symptoms also showed a change in sTREM-1 levels (Pedroso et al., 2020). Our present study demonstrated that increased serum sTREM-1 concentrations were associated with a higher risk of PSCI. There are several possible mechanisms explaining the relationship between sTREM-1 levels and cognitive impairment after an ischemic stroke. First, Xu et al. (2019) found that microglial TREM-1 expression was upregulated following cerebral ischemic injury. By inhibiting TREM-1 with synthetic peptide LP17, neuronal injury may be alleviated and synaptic plasticity may be improved in the hippocampus (Xu et al., 2019). Second, oxidative stress was confirmed to be one of the pathophysiological mechanisms of cognitive impairment after ischemic cerebrovascular disease (Jurcau and Simion, 2020). Studies in both vivo and *in vitro* showed that inhibiting TREM-1 could reduce ROS accumulation and increase superoxide dismutase activity (Liang et al., 2020). Additionally, inhibiting TREM-1 might reduce myeloid cell infiltration and matrix metalloproteinase-9 expression (Boufenzer et al., 2015). Matrix metalloproteinases, whose major source was neutrophils, were associated with the disruption of the blood-brain barrier and cognitive impairment (Lassalle et al., 2016; Sarvari et al., 2020). All of these points strongly suggest that TREM-1 mediates PSCI development through its anti-inflammatory and antioxidative properties.

The advantages of our study include sufficient sample size, prospective cohort study nature, and detailed assessment of cognitive function, all of which made it possible to investigate the association between sTREM-1 concentrations and risk of PSCI. However, some limitations of our study should also be acknowledged. First, since the study was conducted in only one stroke center, our results may not be generalizable to other Chinese patients with ischemic strokes. Second, the subjects with serious illnesses or those with aphasia or dementia were excluded from this study, so a selection bias might be inevitable. This could lead to an underestimation of PCI prevalence. Third, as the study was observational, it was not possible to establish a causal link between STREM-1 levels and PSCI. Finally, serum sTREM-1 concentrations were assessed only once post-admission, restricting our ability to investigate the temporal association between sTREM-1 changes and PSCI following stroke.

In conclusion, higher circulating sTRME-1 levels were independently associated with increased risk of PSCI. Our results provide evidence supporting that sTREM-1 plays a vital role in the PSCI prediction. In addition, further studies with large sample sizes are required to evaluate these associations comprehensively.

## Data Availability

The raw data supporting the conclusions of this article will be made available by the authors, without undue reservation.
